# A Machine Learning Approach for Detecting Cognitive Interference Based on Eye-Tracking Data

**DOI:** 10.3389/fnhum.2022.806330

**Published:** 2022-04-29

**Authors:** Antonio Rizzo, Sara Ermini, Dario Zanca, Dario Bernabini, Alessandro Rossi

**Affiliations:** ^1^Department of Social, Political and Cognitive Science, University of Siena, Siena, Italy; ^2^Technische Fakultät, Friedrich-Alexander-Universität Erlangen-Nürnberg, Erlangen, Germany

**Keywords:** eye-tracking, machine learning, Stroop test, classification, attention load, cognitive interference

## Abstract

The Stroop test evaluates the ability to inhibit cognitive interference. This interference occurs when the processing of one stimulus characteristic affects the simultaneous processing of another attribute of the same stimulus. Eye movements are an indicator of the individual attention load required for inhibiting cognitive interference. We used an eye tracker to collect eye movements data from more than 60 subjects each performing four different but similar tasks (some with cognitive interference and some without). After the extraction of features related to fixations, saccades and gaze trajectory, we trained different Machine Learning models to recognize tasks performed in the different conditions (i.e., with interference, without interference). The models achieved good classification performances when distinguishing between similar tasks performed with or without cognitive interference. This suggests the presence of characterizing patterns common among subjects, which can be captured by machine learning algorithms despite the individual variability of visual behavior.

## Introduction

Viewing is a complex activity, involving cognitive aspects, conscious and unconscious. It manifests itself through motor behavior aimed at acquiring salient information in the form of light radiation. When observing static images, this attentive activity exhibits rapid eye movements called saccades, occurring between the so-called fixations. During fixations, the eye remains still and the information is sampled. It is well known that the cognitive load of individual tasks may influence eye movements statistics (Connor et al., 2004; McMains and Kastner, 2009; Mathôt, 2018), and in particular some variables like average fixation duration, saccade length or saccade velocity, among others. For this reason, it seems reasonable to define techniques based on eye-tracking data in order to recognize recurring patterns related to the visual attention and identify the task that the subject is performing (Klingner, 2010; Zagermann et al., 2016). Indeed, it has already been observed that the variation of the attentive load within different tasks affects the eye movements (Castelhano et al., 2009; Tanaka et al., 2019). Previous study show that simple eye-tracking based parameters, such as fixation count, can be used as a reliable and objective measure to characterize the cognitive load during information detection tasks (Debue and Van De Leemput, 2014) or during inquiry-based learning with multimedia scaffolds (Kastaun et al., 2021). However, current approaches focus on standard fixation-based parameters (as in [1] and [2]) and provide little insights about the influence of cognitive load on the dynamics of visual exploration, which could be better described by saccades and, especially, by higher order correlations in visual behavior.

In this work we analyzed the vision behavior of subjects involved in a Stroop test (Stroop, 1935) while performing two different visual tasks, naming and reading, in order to explore possible effects on human attention on such behavior. The exploratory patterns are expressed through variables related to eye fixations and saccadic movements, since they are both influenced by processing difficulty (Pollatsek et al., 1986).

The execution of each task requires different attention loads to the subject: reading is performed as a fast and automatic process, while naming the color of a word is a slow conscious activity, especially when written with an ink color mismatching its semantics (Kahneman, 2011). The delay in naming colors of words reporting unmatched names of the colors has been described as a cognitive interference. This phenomenon is well-known in experimental psychology and several methods have been developed to test and measure it (Jensen, 1965; Dalrymple-Alford and Budayr, 1966; Bench et al., 1993; Scarpina and Tagini, 2017). To this aim, we set up a visual version of the Stroop test during which we recorded the eye movements of 64 subjects, following the experimental protocol defined in Megherbi et al. (2018). The experiment involves two different tasks, defined as Naming and Reading, and two conditions, defined as “With Interference” and “Without Interference.”

The research questions we tried to address in our study are: (1) Is there evidence of the presence of recurrent visual behavioral patterns for different tasks (naming vs. reading) and conditions (with interference vs. without interference)? (2) Is it possible to generate machine learning models which are able to identify in which task or condition the subject is currently involved? And, in the affirmative case, which kind of algorithm will produce the more reliable model?

The paper is organized as follows. The section “Materials and Methods” describes the experimental protocol set up for stimuli presentation and data collection. In the section “Experiments” we provide a detailed description of the data pre-processing, Machine Learning techniques and metrics for evaluation of the results. Finally, in the “Conclusion” we discuss results and suggest possible directions for future works.

## Materials and Methods

### Participants

We recorded eye movements from 64 subjects (32 female and 32 male, average age = 30,2 ± 11,72). They were informed about the procedure and purpose of the study and signed an informed consent. Experimental procedures conformed to the Declaration of Helsinki and the Italian national for conducting psychological experiments. All subjects were students at the University of Siena and reported normal or corrected-to-normal vision.

### Task and Stimuli

**FIGURE 1:**
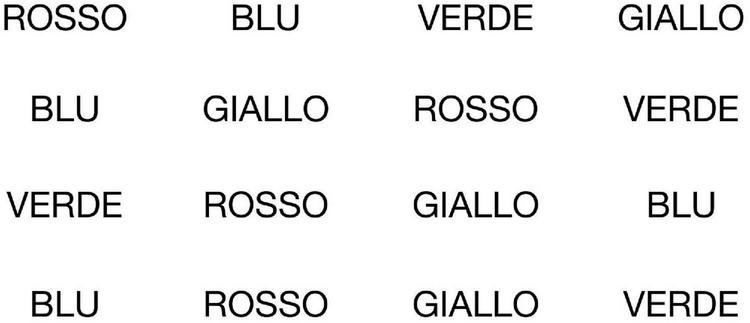
Reading Without Interference (RWoI).

**FIGURE 2:**
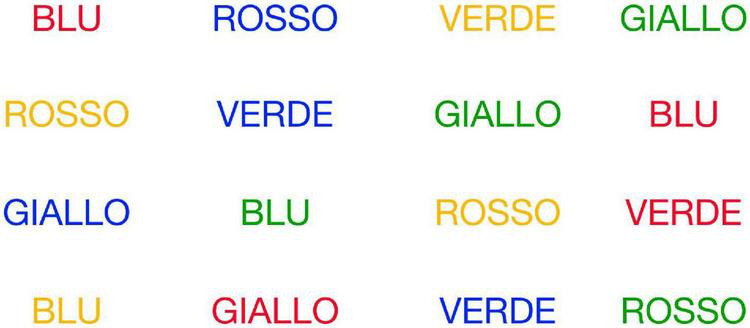
Reading With Interferences (RWI).

**FIGURE 3:**
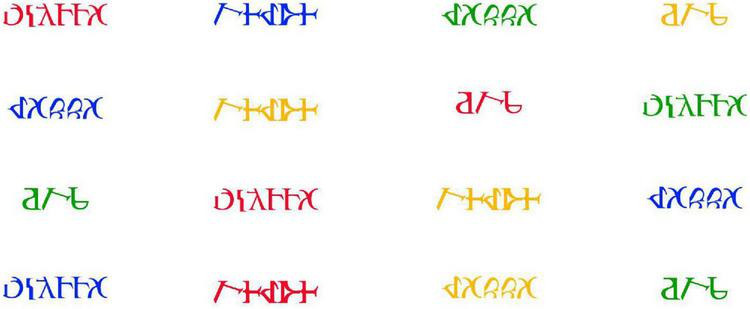
Naming Without Interference (NWoI).

**FIGURE 4:**
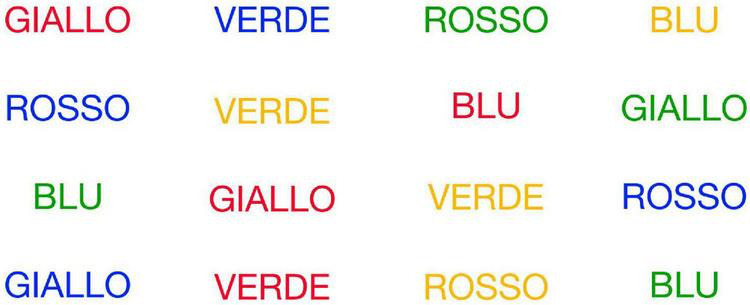
Naming With Interference (NWI).

During the test, the participants had to perform two main tasks: Naming and Reading. These tasks were both divided into two conditions: one “With Interference” and one “Without Interference”; following the experimental protocol defined in Megherbi et al. (2018). The images representing the four stimuli were created by modifying and translating in Italian the ones originally proposed in Megherbi et al. (2018). Stimuli were presented as 1,024 × 768 pixels images, divided in an equally spaced 4 × 4 grid to generate 16 identical cells, representing interest areas. A single word was placed in the center of each cell. The four generated stimuli were composed by:

• Reading Without Interference (RWoI) - Participants had to read the words on screen. The words “ROSSO” (”red”), “GIALLO” (”yellow”), “VERDE” (”green”) and “BLU” (”blue”) were all colored black (see Figure 1).

• Reading With Interferences (RWI) - Participants had to read the words on screen. The words “BLU,” “ROSSO,” “VERDE,” “GIALLO.” etc., were colored red, blue, yellow, green, etc. with a mismatching between the shade used and the meaning of the word (e.g., “ROSSO” was never colored in red) (see Figure 2).

• Naming Without Interference (NWoI) - Participants had to name the color of the words on screen. In this case, the Latin letters were replaced by pseudo-letters constructed to match the real letters’ physical properties (height, number of pixels, and contiguous pixels) by reconfiguring their original characteristics (Megherbi et al., 2018). The pseudo-words were colored red, green, yellow and blue (see Figure 3).

• Naming With Interference (NWI) - Participants had to name the color of the words on screen. The composition of the screen followed the same principles used for the construction of the Reading With Interference (RWI) condition (see Figure 4).

### Procedure

Each of the 64 subjects joined all the four experimental conditions. The presentation order of the conditions were randomized balancing the set of possibilities among participants. Eye movements were recorded by an EyeLink Portable Duo1 (2022) set to 500 Hz sampling rate and in Head-Free mode. Images were presented on a 17 inches display (1920 × 1080 pixels), placed perpendicularly in front of the participant at a distance from the eyes ranging in 46-52 cm. The light in the room and ambient noise were under control so to keep such parameters constant along all the experimental sessions, Written instructions were first presented to the participants, followed by an oral brief aimed at assessing the proper understanding of the test and the associated procedure. The experiment was preceded by an initial unrecorded trial and a standard 5-point calibration. Between the instructions and the stimulus screens, a white screen containing a circular trigger located at the top-left corner of the task image was presented. Each trial began when the participant fixated the trigger for at least 100 ms. The trial was completed when the participant pressed the “space” key. During the execution, the experimenter annotated on an Excel spreadsheet any relevant information regarding the experience of the subject and possible technical issues (e.g., if the first calibration failed). Three participants unable to correctly read words and instructions on the screen were discarded. All the subjects performed accurately in the four tasks, with few errors only in the Naming with Interference conditions. The experimental session was concluded by a debrief session where the participants reported their subjective impressions about the task performed.

### Eye Tracking Features

The Eye Tracking features adopted as Dependent variables (e.g., Fixation position, fixation time, etc.) about eye movements were extracted from two of the reports generated by the software released with the eye-tracker device (The EyeLink Data Viewer). The first report used was the one about fixations, listing, for each trial, the list of fixations computed by the software together with correspondent time point, xy position, duration and area of interest (a rectangle around each one of the words. The second one listed all the recorded saccades during each trial, reporting xy starting and ending point, amplitude, velocity, direction and duration. The two reports are used to compute statistical features about fixations and saccades. However, we found that a fine-tuning process was necessary to improve the data quality. Fixations that fall well outside of the areas of interest were discarded, since they could be either due to an instrumental artifact or to a subject’s activity not related to the task. Gaze data referring to the head and tail of the experiment were also discarded. We refer to the head of a trial as the time until the trigger was activated. Indeed, the trigger dot was actually introduced to ensure similar initial conditions among subjects. In an analogous way, we refer to the tail of a trial as the time between the observation of the last word (the bottom right one) and the push of the “space” key, which concludes each trial. We empirically found that removing the last five fixations guarantees a good noise cleaning without filtering out any relevant fixations. A fixation threshold was used to discard fixations which are too short or too long, which could be due to noisy gaze points reads or to approximation errors introduced by the software. It is well known that meaningful fixations during reading tasks are within the range 100-400 ms (Liversedge and Findlay, 2000; McConkie and Dyre, 2000; Majaranta and Räihä, 2002). Because of the specific tasks under investigation, we adopted a more specific method to select fixations of interest. Since fixations of interest for reading tasks are considered around 200-250 ms, we dropped fixations below 200 ms. For the longest ones, we applied an outlier detection method, to select those samples with z-score (Devlin et al., 1975) lower than 3. This approach appeared to be empirically valid, since it allowed us to keep the maximum fixation duration in the interval of 800-1200 ms within subjects and experiments. This range is sensibly higher than the ones proposed in literature, but we avoided the use of a constant threshold in order to guarantee an additional degree of freedom so as to include the maximum duration of the fixations in a trial as one of the variables of interest.

For each subject, and for each condition (NWI, NWoI, RWI, RWoI), a set of features related to eye movements were extracted. All of them were considered as dependent variables in respect to the four experimental conditions. Seven dependent variables were related to the fixation of the glance, instead other 22 dependent variables were related to the movement of the glance (saccade).

The 7 dependent variables related to the fixation of the gaze:

• Number of fixations: total number of fixations (**n_fix**).
• Average fixation length: the average of the duration among all the fixations (**fix_max, fix_mean**).
• Maximum fixation length: the maximum of the duration among all the fixations (**norm_fix_max, norm_fix_mean**).
• Horizontal/Vertical regressions: the number of times that the eyes step backward in their horizontal/vertical path (assumed left to right and up to down, respectively), excluding the changes of line in the horizontal counting (**x_regressions, y_regressions**).

The 22 dependent variables related to the movement of the gaze (saccade):

• Up/Down/Left/Right Frequency: the counting of saccadic movements in each direction, normalized by the total number of saccades (**up_freq, down_freq, left_freq, right_freq**).
• Minimum/Average/Maximum saccade duration: statistics about the duration of each saccade (**min_duration, avg_duration, max_duration**).
• Minimum/Average/Maximum saccade velocity: statistics about the estimated velocity of each saccade (**min_vel, avg_vel, max_vel**).
• Minimum/Average/Maximum saccade amplitude: statistics about the amplitude of each saccade - in degrees of visual angle (**min_ampl, avg_ampl, max_ampl**).
• Minimum/Average/Maximum saccade angle: angle between the horizontal plane and the direction of the next saccade (**min_angle, ave_angle, max_angle**).
• Minimum/Average/Maximum saccade distance: statistics about the distance of each saccade - in degrees of visual angle (**min_distance, avg_distance, max_distance**).
• Minimum/Average/Maximum saccade slope: statistics about the slope of each saccade with respect to the horizontal axis (**min_slope, avg_slope, max_slope**).

All the scripts and functions used to process the data are implemented in Python v3.7.5 (Van Rossum and Drake, 2009), using Pandas v0.25.3 (McKinney, 2010), Scikit-learn v0.21.3 (Pedregosa et al., 2011) and SciPy v1.3.1 (Virtanen et al., 2020).

## Results

The results are reported into two separated branches. The first concerns the traditional inferential analysis carried out through a comparison between conditions by means of an Analysis of Variance. The second concerns an analysis of the data carried out with a selection of Machine Learning algorithms aimed at developing models that could predict the specific experimental conditions starting from the data collected.

### Statistical Analysis

All 29 dependent variables were used to make a comparison between: (1) Naming vs. Reading in condition of Not Interference (i.e., NWoI vs. RWoI); (2) Naming Without Interference vs. Naming With Interference (i.e., NWoI vs. NWI); and (3) Reading Without Interference vs. Reading With Interference (i.e., RWoI vs. RWI) by means of a standard one-way ANOVA (Fisher, 1992) using the SciPy implementation (Virtanen et al., 2020) in Python v3.7.5 (Van Rossum and Drake, 2009). Since the difference between Naming and Reading tasks is well documented in literature (e.g., Kahneman, 2011), the first test was considered a control condition for the whole experiment. Furthermore, we made the comparison between Naming and Reading both for the condition without interference (see column NWoI vs. RWoI in Table 1) and by putting together the conditions with interference with those without interference (see column Naming vs. Reading in Table 1) in order to see if the trend of results is consistent as it appear to be.

**TABLE 1:** *P*-values generated by the one-way ANOVA on fixations variables when comparing three pairs of tasks: NWoI vs. RWoI, NWI vs. NWoI, and RWI vs. RWoI.

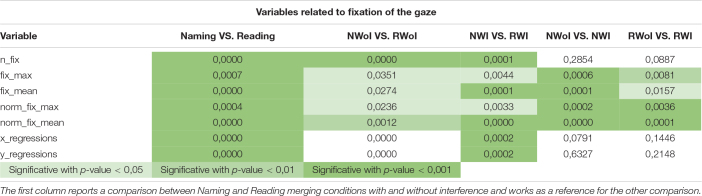

The second and third comparisons are the focus of the presented work, carried out in order to assess if different attention levels, required to perform the two different tasks (Naming and Reading), can be caught by variables related to eye movements. In Table 1 we report the significance values p obtained for in each comparison involving the variables about fixations. As we can see, the differences between Naming and Reading tasks are well represented by statistics about the duration of fixations (both average and maximum). In the second test, the effects of interference in Naming is highly expressed by the number of fixations and the eye regressions in both axes.

A different pattern of results is obtained taking into consideration the variables related to saccadic movements. Looking at Table 2, it is possible to observe produced a much less clear pattern of results. Indeed, a high level of significance (*p* = 0.005) was achieved only by the variables Average and Maximum Saccades Duration when comparing NWI vs. NWoI, and in a few other cases we obtained a significant difference.

**TABLE 2:** *P*-values generated by the one-way ANOVA on saccade related variables when comparing three pairs of tasks: NWoI vs. RWoI, NWI vs. NWoI, and RWI vs. RWoI.

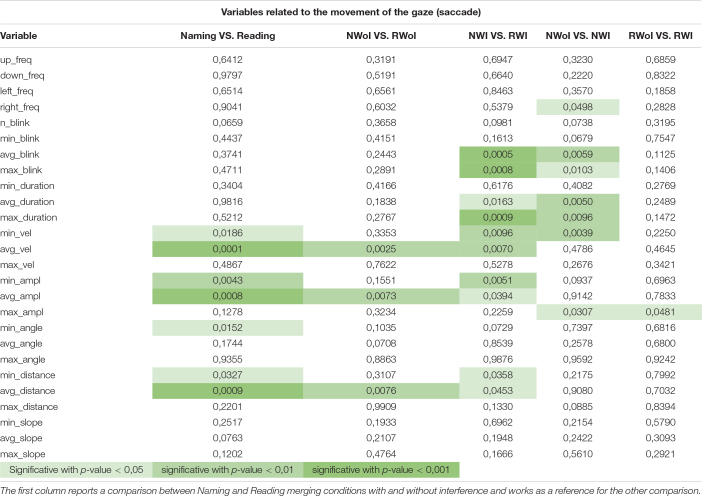

### Machine Learning Analysis

Given these results, none of the variables produce, by itself, a satisfactory task characterization. Moreover, this kind of statistics does not directly provide a predictive model with good generalization performances when we need to infer new knowledge on unseen data. Indeed, a threshold model achieves poor performances, probably because of the high inter-subjects variability. Our claim is that more complex behaviors involving the dynamics of the attentive process or task specific gaze strategies can be captured by more complex (non-linear) models. Such models can take into account non-linear interactions among variables, possibly represented by means of hidden representations, partially overcoming the high variability. In the context of hypothesis testing, we applied Machine Learning techniques to assess statistical significance through a dual approach in which we evaluated the performances of selected learning models in classification tasks between two populations that we assume to be distinct (Mjolsness and DeCoste, 2001; Oquendo et al., 2012; Vu et al., 2018). We applied four different machine learning techniques and evaluated the performances achieved on the collected dataset. Our goal is to assess if the cognitive interferences that affect the gaze dynamics can be detected by machine learning algorithms exploiting eye-related features. Eventually, we would like to find out that classification performances are consistently better than a random baseline in order to support the hypothesis that the two populations are intrinsically distinct. Our aim is not to find the best machine learning techniques but to observe how four of the basic learning algorithms behave in their classification task.

Since the four stimuli are presented to each subject, our dataset consists of 64 examples per class, which could be too small to capture complex dynamics. However, to partially address this inter-subject variability, we exploited an *ad hoc* normalization technique. For each subject, we computed the mean of each variable within the four tasks, and subtracted it to the original values. This mitigates individual effects on each task, and improves the final representativeness of the variables. We are aware that the sample size is limited for a machine learning study and therefore limited the complexity of the chosen methods. Indeed, more advanced machine learning methods, such as deep learning, can allow modeling of more complex phenomena, but they are notably data hungry and are not suitable for the current study. However, with respect to the machine learning algorithm adopted, we claim that the reliability of the results is based on cross validation, which guarantees unbiased estimation of the models’ performance on unobserved data.

We repeated the same tests investigated within the statistical analysis by setting up three separated binary classification tests: NWoI vs. RWoI, NWI vs. NWoI, and RWI vs. RWoI. We avoid a global 4-class test since the dynamics of the tasks are too complex to be modeled by such a small number of samples. We exploited the Scikit-learn (Pedregosa et al., 2011). Python software package to test the four different classifiers (Bishop, 2006):

(i). Random Forests (RF) are an ensemble of decision trees based on bootstrapping. Different models are trained on a subset of samples and the final decision is taken by majority voting.
(ii). Logistic Regression (Logistic) is a statistical model that in its basic form uses a logistic function, applied to a weighted average of the input features, to model a binary dependent variable (the model prediction).
(iii). Artificial Neural Networks (ANN) are a well known class of learning algorithms inspired by the biological neural networks; they are based on a collection of units or nodes, called artificial neurons, connected by edges which represent the flow of information; edges are in fact numbers and represent the parameters of the model, typ- ically learned by the back-propagation of an error signal with respect to the target.
(iv). SupportVectorMachines(SVM) are supervised learning models used for binary classification. SVMs can learn non-linear separation surfaces by means of the so-called kernel trick, implicitly mapping their inputs into high- dimensional features space.

**FIGURE 5:**
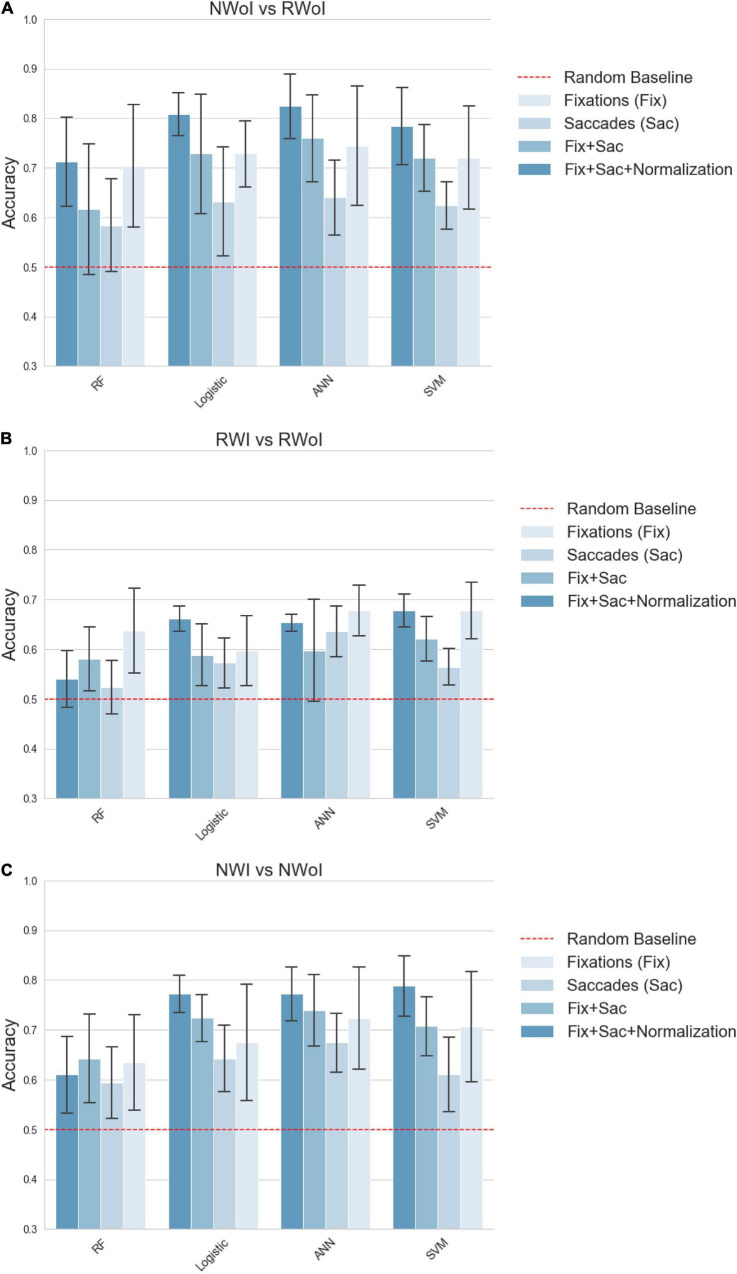
Classification performances in terms of **Accuracy**. **(A)** Naming without Interference vs Reading with Interference. **(B)** Reading with Interference vs Reading without Interference. **(C)** Naming with Interference vs Naming without Interference. Each plot represents a different binary classification task, indicated in the title. Bars indicate the average Accuracy on a 5-fold cross validation setting, while the confidence interval represented by black lines on the top of each bar indicates the standard deviation. Each group of contiguous bars refers to the performance achieved by the same classifier: RF, Logistic, ANN and SVM. Bar’s color indicates the set of features fed as input to the classifier: *Fix* (features related to fixations), *Saccades* (features related to saccades), *Fix* + *Saccades* (features related to fixations and saccades), *Fix* + *Saccades-norm* (features related to fixations and saccades with subject-wise normalization). The dashed red line sets the reference of the random baseline 0.5.

**FIGURE 6:**
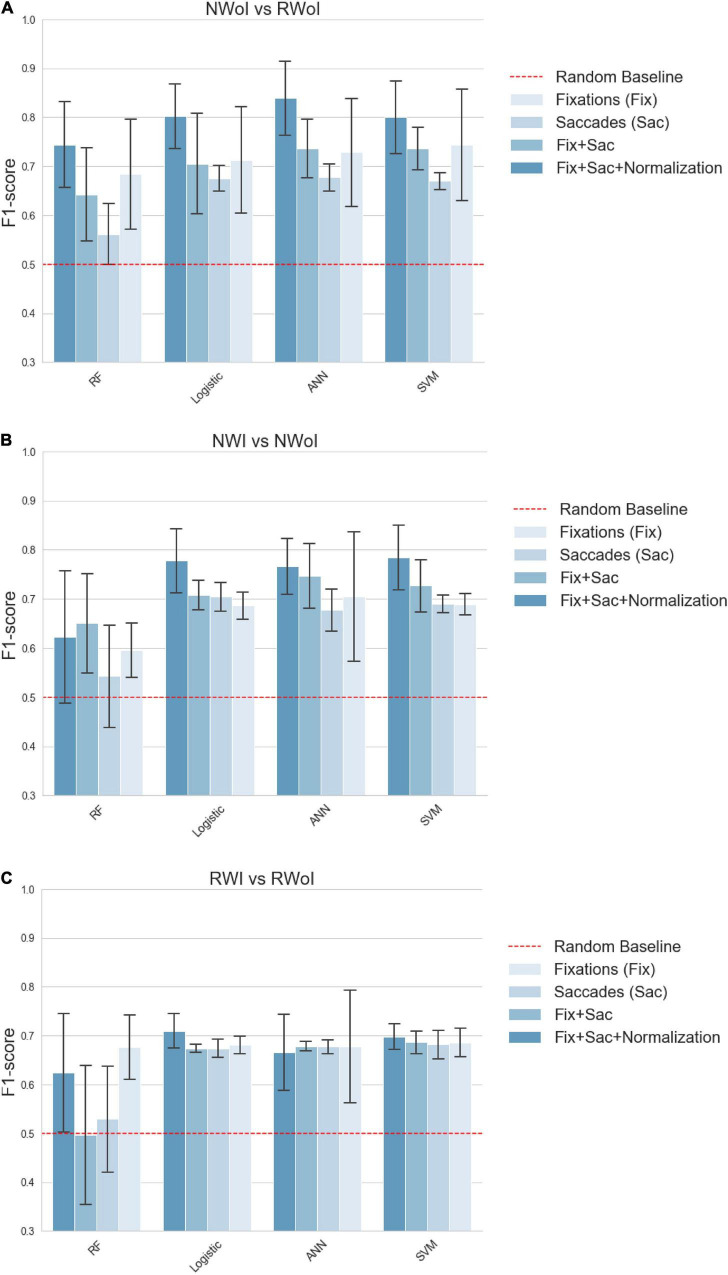
Classification performances in terms of **F1-score**. **(A)** Naming without Interference vs Reading with Interference. **(B)** Reading with Interference vs Reading without Interference. **(C)** Naming with Interference vs Naming without Interference. Each plot represents a different binary classification task, indicated in the title. Bars indicate the average F1-score on a 5-fold cross validation setting, while the confidence interval represented by black lines on the top of each bar indicates the standard deviation. Each group of contiguous bars refers to the performance achieved by the same classifier: SVM, RF, ANN, and Logistic. Bar’s color indicates the set of features fed as input to the classifier: *Fix* (features related to fixations), *Saccades* (features related to saccades), *Fix* + *Saccades* (features related to fixations and saccades), *Fix* + *Saccades-norm* (features related to fixations and saccades with subject-wise normalization). The dashed red line sets the reference of the random baseline. For both parameters (Accuracy and F1 -score) all the classifiers and features pairs are significantly above the random baseline 0.5, and in best cases above 0.8, with a similar but not identical pattern of results between Accuracy and F1-score.

We performed a 5-fold Cross- Validation for each classifier and computed the average of achieved Accuracy (see Figure 5) and F1-scores (see Figure 6). This should guarantee that results do not depend on the choice of the test set, even if the relatively high variability presented depends on the small size of the test (one single sample which is not classified correctly heavily affects the results). All the tests were implemented in Python v3.7.5 (Van Rossum and Drake, 2009) using the Scikit-learn v0.21.3 (Pedregosa et al., 2011) implementation of Cross-validation and of the tested Machine Learning algorithms. Plots have been generated in Seaborn v0.9.0 (Waskom, 2021).

To improve the performances of Machine Learning algorithms, features were normalized in [0,1] (we found this method to slightly outperform z-normalization in our case). In addition, to investigate more in depth the information expressed by the features, we generated four sets of variables that we tested independently.

• Fix. It was composed by the variables extracted from fixations, when filtering out fixations shorter than 200 ms.
• Saccades. It was composed of variables extracted from saccades, aimed at capturing gaze dynamics and visual ex- ploration schemes.
• Fix + Saccades. It is composed both from fixations and saccades features.
• Fix + Saccades-norm. Since variables related to eye- movements are characterized by a strong inter-subject variability, for each subject we computed and subtracted the mean of each variable through the different experiments. This process aimed at shifting the mean of the distribution of each variable around zero for each subject with the idea of simplifying the comparison of the different conditions among different subjects.

## Discussion

The results obtained considering as dependent measure the features related to the fixation of the glance appear to be in agreement with the literature, since saccades regressions (see x_regressions, y_regressions in Table 1) are found to be more frequent and larger when the reader encounters some difficulties (Pollatsek et al., 1986; Murray and Kennedy, 1988). The results in these first two tests were also confirmed by the subject’s report in the debrief session, in which they admitted to perceive the Naming task as counterintuitive, especially in presence of interference. On the other hand, they confirmed to perceive the Reading task as trivial, with little additional difficulty introduced by interference. This perception is also in agreement with our results, since the p-values for the RWI vs. RWoI are in general higher with respect to the other tests. Each of 7 features related to the fixation of the glance appear to be a good indicator for distinguishing between Naming vs. Reading task. Instead only the features related to the Average fixation length (**fix_max, fix_mean**) and that related to the Maximum fixation length (**norm_fix_max, norm_fix_mean**) showed a significant difference between the two tasks (Naming and Reading) with and without interference. Thus the features associated to the fixation of the glance appear to be good indicators of the different task. And the information associated with fixation length is crucial to distinguish a situation with cognitive conflict from one without conflict. This provides an answer to our first research question, namely: the presence of recurrent visual behavioral patterns for different tasks (naming vs. reading) and conditions (with interference vs. without interference).

Instead the features related to the movement of the glance presents a different scenario. That is, there are no dependent variables that consistently allow us to distinguish between Tasks or Conditions. According to this kind of analysis it seems that the movements of the eye (in respect to the fixation of the eye) are not a potential indicator of the task, nor of the cognitive conflict. These results corroborated the hypothesis that attention level influences gaze behavior but, apparently, only for what concerns fixations related variables. Indeed, previous study focuses on standard fixation-based parameters (e.g., Debue and Van De Leemput, 2014; Kastaun et al., 2021) and provides not much insights about the influence of cognitive load on the dynamics of visual exploration, which could be better described by saccades and higher order correlations in visual behavior (see below).

Considering our second research question, if it is possible to generate machine learning models able to identify in which task or condition the subject is currently involved, it seems that such connections can be captured by an automatic classifier even at a small scale (i.e., with few training samples). Interestingly, a global trend is observed while considering different sets of input features. Combining features about fixations and saccades brings an improvement on the performances of each classifier, compared with the case in which separated features are exploited (see the results associated with saccade’s parameters in the ANOVA tests). This result also connects the attentive load to different exploration strategies of the visual scene. As already noticed, information about backward saccades, are connected to more complex types of reasoning, typical of attentive processes, which are led by a need re-analysis or re-sampling already visited portions of the scene (Pollatsek et al., 1986; Murray and Kennedy, 1988). Moreover, a strong improvement is achieved by applying a subject-wise normalization. This confirms that the analyzed scenario is highly affected by personal behaviors, but we showed that these effects can be mitigated by the application of standard statistical techniques. Finally, we could observe that the Random Forests achieved performances which are considerably worse with respect to the other algorithms, sometimes even close to the random baseline. This could be due to the fact that the decision tree is unable to extract high-level correlations among variables, but most of all that the random sub-sampling negatively emphasizes the inter-subject variability.

## Conclusion

The experimental results supported the hypothesis) that different attentive loads present recurrent visual behaviors that can be characterized by a statistical analysis of variables related to eye fixations. Furthermore, these patterns can be modeled (hypothesis 2) by data-driven Machine Learning algorithms which are able to identify, with reasonable accuracy, the different conditions in which individuals are involved. We show that situations of cognitive conflict are captured by the gaze data and the related statistical analysis. It is worthwhile to note that the combining of features related to both fixations and saccades increases the accuracy of the classifiers while the features related to the saccades alone are not enough to distinguish the condition with cognitive interference from that without interference. This suggests that subjects, among different tasks, use to implement task-specific schemes to regulate their gaze dynamics. We found that the exploited normalization techniques are useful when addressing wide inter-subject variability to improve the comparison among different individuals. However, these issues could be addressed more effectively by a large scale data collection to obtain more versatile Machine Learning models and more reliable results. At the same time it would be worthwhile to compare the gaze behavior in the Stroop task with the gaze behavior of other tasks that produce cognitive conflict such as the Simon task (Lu and Proctor, 1995; Dolk et al., 2014). The investigation carried out with machine learning models could contribute to the debate if the interference effects occur at different processing stages and are or not attributable to different mechanisms (Scerrati et al., 2017). In particular it could shed some light on the role of a motor component, namely glance behavior, that is non-considered in the debate between the Perceptual account and the Decision account of cognitive conflicts. Besides this, future research directions could include the integration in the analysis data related to pupillary response, since they are already proven to be connected to attentive and cognitive load (Klingner, 2010; Mathôt, 2018). This could help to explain more in depth connections among visual attention and eye movements, but also to develop more robust practical scenarios. Indeed, similar analysis turn out to be useful in applications such as monitoring attentive state of drivers (Palinko et al., 2010) or understanding truth telling and deception (Wang et al., 2010).

## Data Availability Statement

The raw data supporting the conclusions of this article are available at this link https://tinyurl.com/EyeTrackData.

## Ethics Statement

Ethical review and approval was not required for the study on human participants in accordance with the local legislation and institutional requirements. The patients/participants provided their written informed consent to participate in this study. Written informed consent was obtained from the individual(s) for the publication of any potentially identifiable images or data included in this article.

## Author Contributions

ARi, SE, DB, and ARo conceived and designed the study. SE and DB contributed to the data collection. ARo and DZ conducted all analyses. ARi, SE, DB, ARo, and DZ wrote the manuscript. All authors contributed to the revision of the article and approved the submitted version.

## Conflict of Interest

The authors declare that the research was conducted in the absence of any commercial or financial relationships that could be construed as a potential conflict of interest.

## Publisher’s Note

All claims expressed in this article are solely those of the authors and do not necessarily represent those of their affiliated organizations, or those of the publisher, the editors and the reviewers. Any product that may be evaluated in this article, or claim that may be made by its manufacturer, is not guaranteed or endorsed by the publisher.

## Supplementary Material

The supplementary material for this article concerns copy of the stimuli used, pictures of the experimental setting and a short video of the initial set up.
