# Supplementary figures and images for: A Machine Learning Approach for Detecting Cognitive Interference Based on Eye-Tracking Data
Source: Front Hum Neurosci. 2022 Apr 29;16:806330. doi: 10.3389/fnhum.2022.806330 (PMC9101480; doi:10.3389/fnhum.2022.806330)

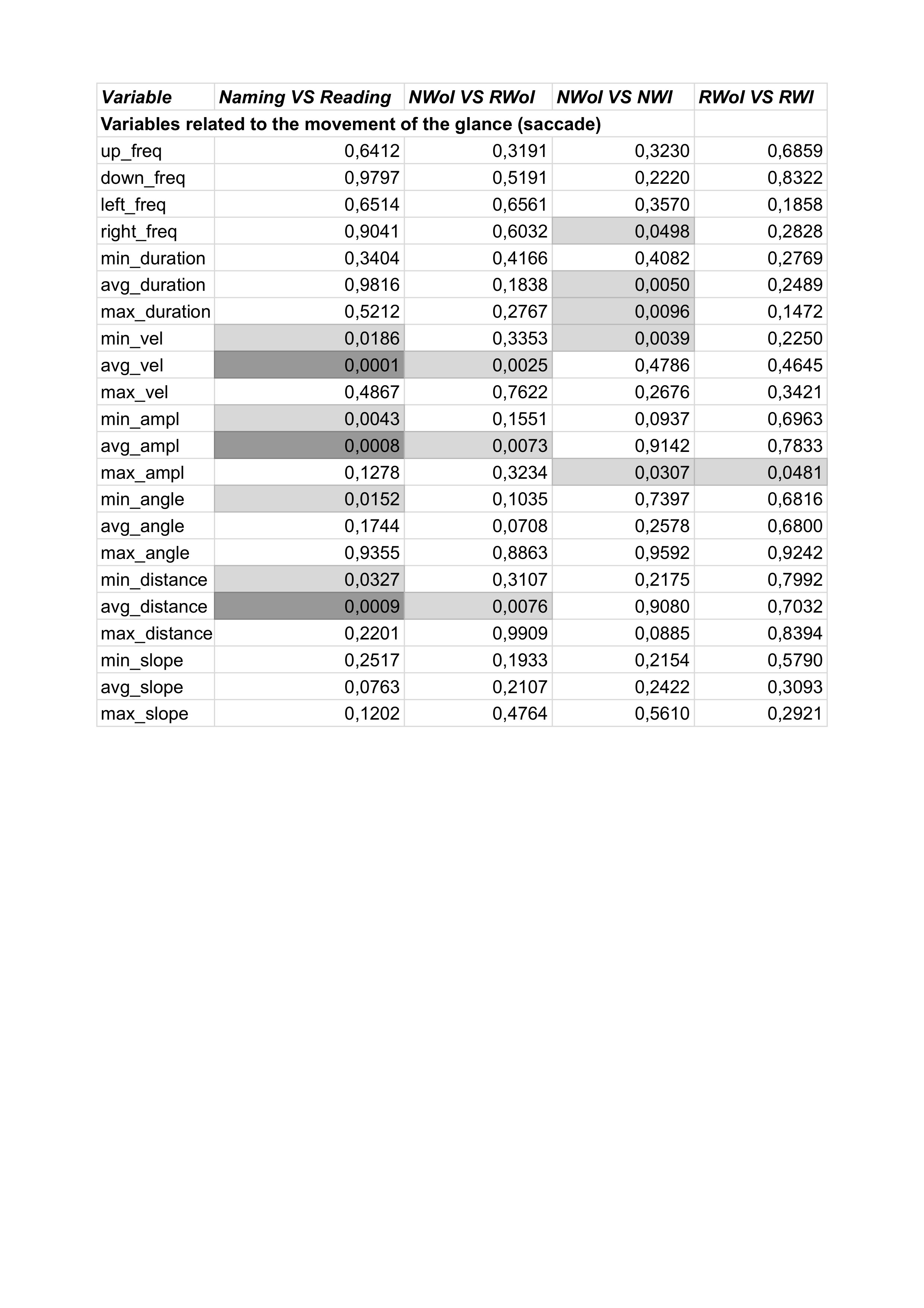

Supplement: Supplementary file 2 [file Image_1.JPEG]

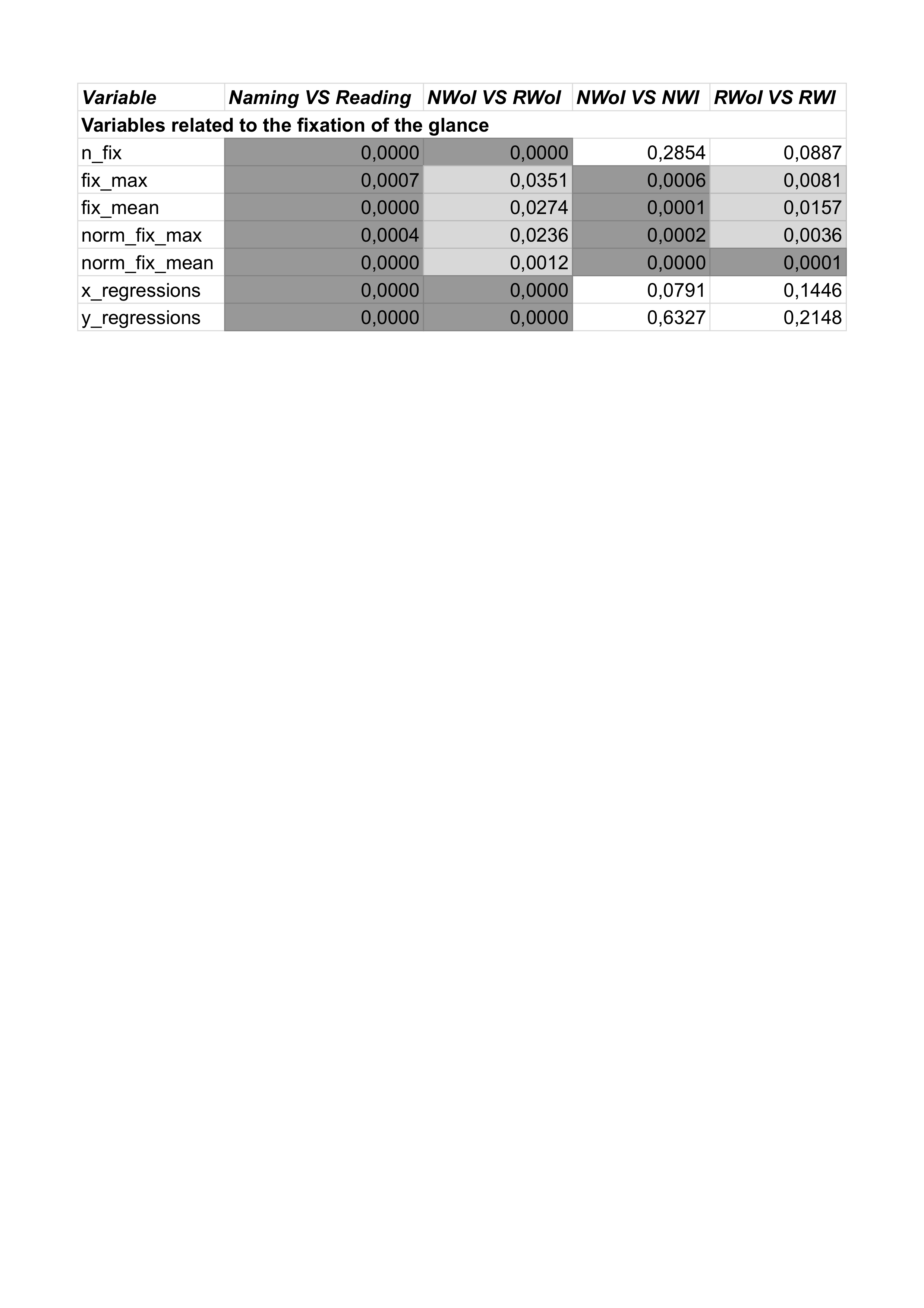

Supplement: Supplementary file 3 [file Image_2.JPEG]

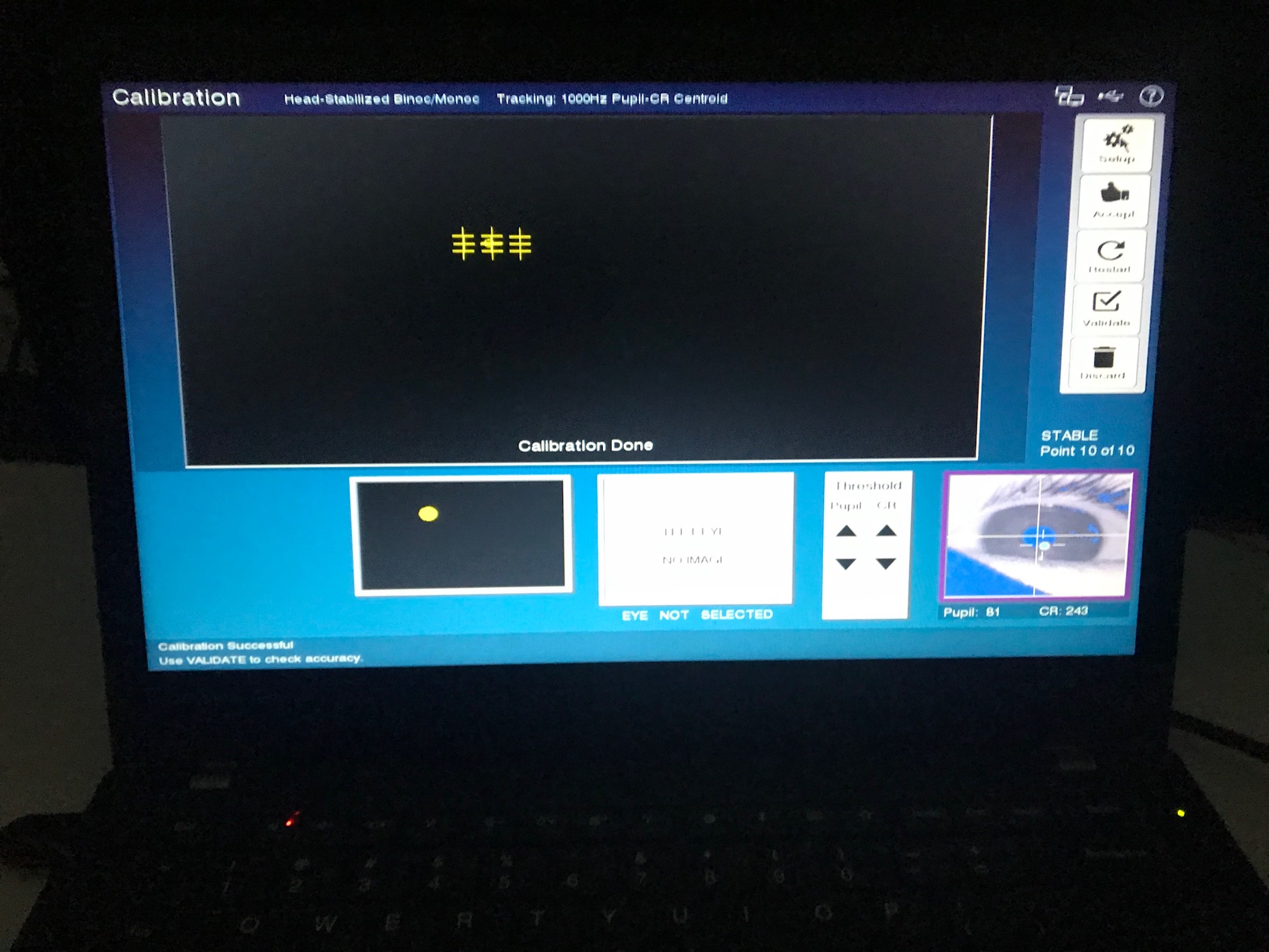

Supplement: Supplementary file 4 [file Image_3.jpeg]

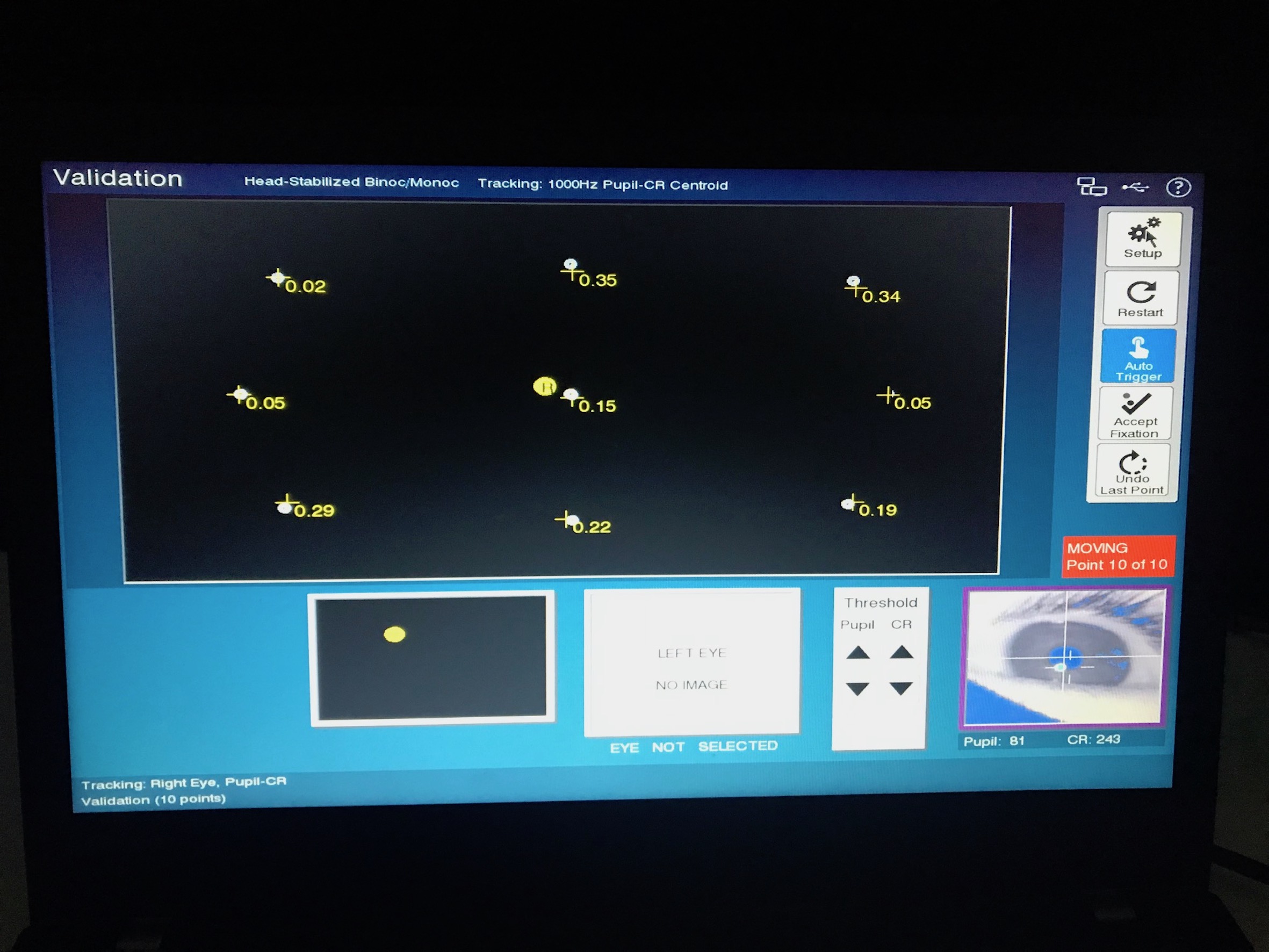

Supplement: Supplementary file 5 [file Image_4.jpeg]

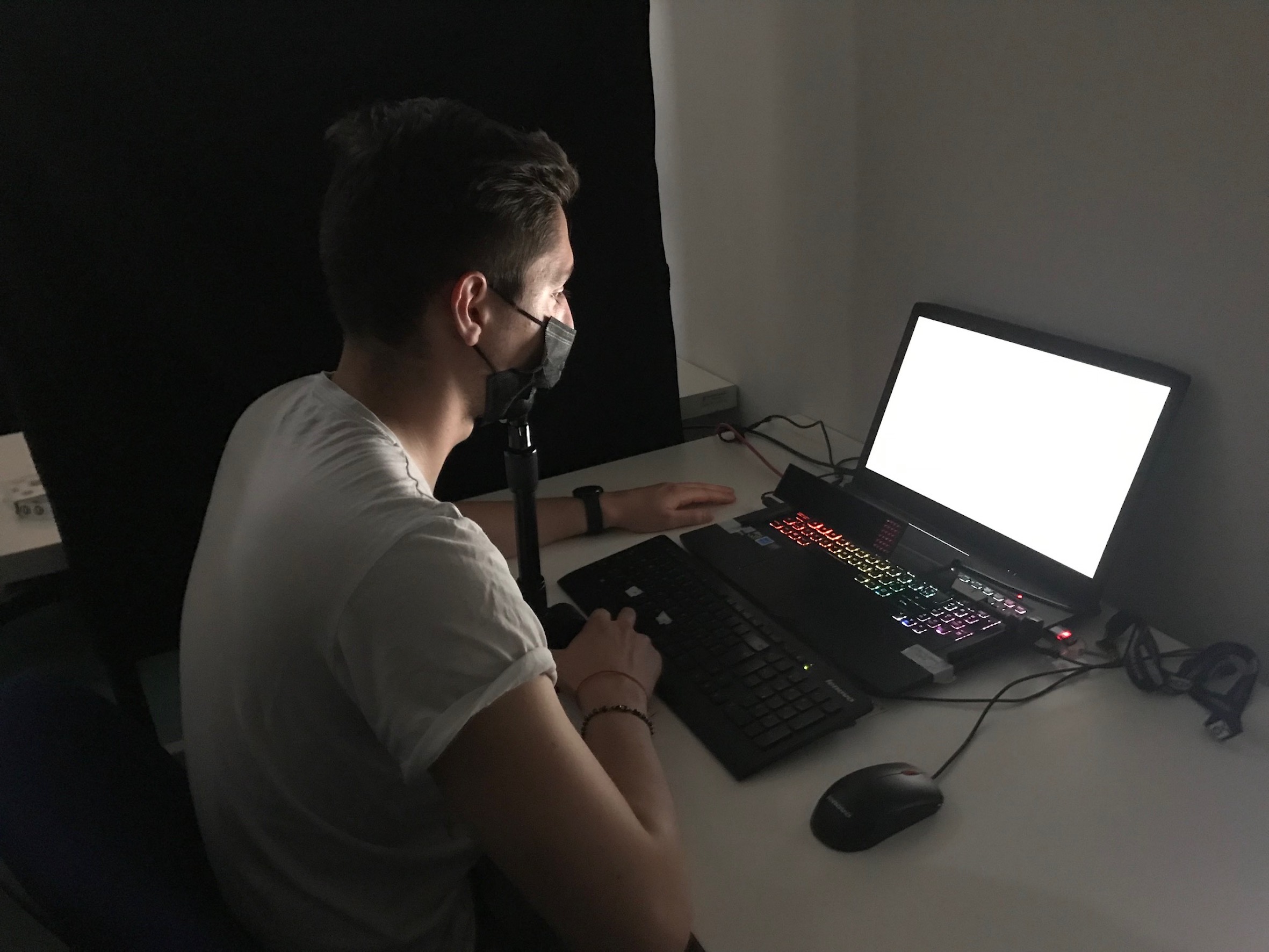

Supplement: Supplementary file 6 [file Image_5.jpeg]
